# Dynamic Subcellular Localization of Iron during Embryo Development in Brassicaceae Seeds

**DOI:** 10.3389/fpls.2017.02186

**Published:** 2017-12-22

**Authors:** Miguel A. Ibeas, Susana Grant-Grant, Nathalia Navarro, M. F. Perez, Hannetz Roschzttardtz

**Affiliations:** ^1^Departamento de Genética Molecular y Microbiología, Pontificia Universidad Católica de Chile, Santiago, Chile; ^2^Departamento de Ecología, Pontificia Universidad Católica de Chile, Santiago, Chile

**Keywords:** embryo development, iron, Brassicaceae, nucleus, perls/DAB

## Abstract

Iron is an essential micronutrient for plants. Little is know about how iron is loaded in embryo during seed development. In this article we used Perls/DAB staining in order to reveal iron localization at the cellular and subcellular levels in different Brassicaceae seed species. In dry seeds of *Brassica napus, Nasturtium officinale, Lepidium sativum, Camelina sativa*, and *Brassica oleracea* iron localizes in vacuoles of cells surrounding provasculature in cotyledons and hypocotyl. Using *B. napus* and *N. officinale* as model plants we determined where iron localizes during seed development. Our results indicate that iron is not detectable by Perls/DAB staining in heart stage embryo cells. Interestingly, at torpedo development stage iron localizes in nuclei of different cells type, including integument, free cell endosperm and almost all embryo cells. Later, iron is detected in cytoplasmic structures in different embryo cell types. Our results indicate that iron accumulates in nuclei in specific stages of embryo maturation before to be localized in vacuoles of cells surrounding provasculature in mature seeds.

## Introduction

Nutrient reserves in the seed must be sufficient to sustain plant establishment until the root system has developed enough to provide nutrients from the soils. High nutrient content of seeds is particularly important for plants growing in unfavorable nutritional conditions and has been related to higher seed viability and seedling vigor. Besides its impact on plant growth, nutrient levels in seeds are an important consideration for human and/or livestock seed-based nutrition (Roschzttardtz et al., 2017).

Iron is an essential micronutrient for plant growth and development. Despite its importance, the prevalent low iron bioavailability in the soils of main agricultural areas of the world limits plant productivity, fertility, and germination rates (Guerinot and Yi, 1994). As a consequence, iron contents in seeds is diminished which results in negative impacts in human and animal health, since seeds are a main source of food for humans and animals (Fan et al., 2008; DeFries et al., 2015). In humans, iron deficiency in women and children under 2 years is a serious and growing public health problem and a major concern for the World Health Organization. Therefore, understanding seed iron distribution and storage at the physiological and molecular level is key to design biotechnological applications to improve iron content of staple seeds.

Different approaches have been used in order to study where iron localizes in plant organs and at subcellular level. In plant, iron accumulates in different compartments depending of the tissue and cell type. Among these are apoplast in root vasculature, plastids in leaves and pollen grain, vacuoles in embryos, and nuclei in different cell types (Roschzttardtz et al., 2009, 2011, 2013; Lanquar et al., 2010).

Regarding seeds, it has been described that iron accumulates in embryo during maturation stage of seed development (Roschzttardtz et al., 2009). So far, VACUOLAR IRON TRANSPORTER1 (VIT1) is the only player described involved in the iron loading into the vacuoles of endodermis cell layer in Arabidopsis embryos. Mutants in *VIT1* mislocalize iron, which accumulates in vacuoles of cortex cells in hypocotyl and subepidermal cells of abaxial side of cotyledons (Kim et al., 2006; Roschzttardtz et al., 2009; Eroglu et al., 2017). Interestingly, *vit1* mutants do not grow correctly in iron deficient conditions, suggesting that iron localization in seed has an important role in seed physiology. In the absence of functional VIT1 or during germination, the manganese and iron transporter MTP8 is responsible for loading iron in the vacuoles of subepidermal cells of cotyledons (Eroglu et al., 2017).

Little is known about the mechanism involved in iron loading in embryo during seed development. Recently Grillet et al. (2014) described that ascorbate efflux from Pea embryos is required for iron loading in seeds.

In this article we used, as model plants, species belonging to the Brassicaceae family in order to describe the subcellular compartments where iron accumulates in embryo during seed development. One of our models, *Brassica napus* L., is one of the most important oil crops almost all over the world. The tetraploid *B. napus* is a hybrid derived from *Brassica oleracea* and *Brassica rapa* and it shares more than 86% similarity in protein coding sequence with *Arabidopsis thaliana* (Cavell et al., 1998; Parkin et al., 2005). Furthermore, rapseed is not only important for vegetal oil, but also a major source for industrial materials such as biofuel (Chen et al., 2017).

Our results show that iron is accumulated first in nuclei of integument cells, endosperm and embryo cells in the early maturation stage of seed development before being located in vacuoles of endodermis cells. Some differences in iron localization were also observed in mature embryos of *Brassica napus*, *Nasturtium officinale*, *Lepidium sativum*, *Brassica oleracea* and *Camelina sativa* compared with *Arabidopsis thaliana*.

## Materials and Methods

### Plant Material and Growth Conditions

*Brassica napus, Nasturtium officinale, Camelina sativa*, *Lepidium sativum*, and *Brassica oleracea* seeds were purchased at a local market. *B. napus* and *N. officinale* plants were grown on soil in a greenhouse at 23°C under long-day conditions (16-h/8-h day/night cycle).

### Histochemical Staining of Iron with the Perls/DAB Procedure

Iron staining was performed according to Roschzttardtz et al. (2009). *Brassica napus, Nasturtium officinale, Camelina sativa, Brassica oleracea*, and *Lepidium sativum* dry seeds or seeds from *B. Napus* and *N. officinale* at different developmental stage were vacuum infiltrated with fixation solution (2% w/v paraformaldehyde in 1 mM phosphate buffer pH 7.0) for 45 min and incubated for 16 h in the same solution. The fixated seeds were dehydrated with ascendant concentration of ethanol (50, 70, 80, 90, 95, and 100%), later were incubated 12 h with a solution of butanol/ethanol 1:1 (v/v), and finally were incubated 12 h with 100% butanol. Then, the seeds were embedded in the Technovit 7100 resin (Kulzer) according to the manufacturer’s instructions and thin sections (3 μm) were cut and were deposited on glass slides. For Perls/DAB staining, slides were incubated with 2% (v/v) HCl and 2% (w/v) K-ferrocyanide (Sigma–Aldrich) for 45 min For the DAB intensification, each glass slide was washed with distilled water, later were incubated in a methanol solution containing 0.01 M NaN_3_ (Sigma–Aldrich) and 0.3% (v/v) H_2_O_2_ (Merck) for 1 h, and then washed with 0.1 M phosphate buffer (pH 7.4). For the intensification reaction, the glass slides were incubated between 10 and 30 min in a 0.1 M phosphate buffer (pH 7.4) solution containing 0.025% (v/v) H_2_O_2_, and 0.005% (w/v) CoCl_2_^∗^6H_2_O (intensification solution). To stop the reaction each slide was rinsed with distilled water.

### Iron Quantification

A microwave-assisted acid digestion was performed using 6.0 mL of concentrated HNO_3_ (Winkler) and 1.0 mL of 30% (v/v) H_2_O_2_ added over 10–20 mg of seeds of each genotype. The colorless digestate were filled up to 15 mL using deionized water. The iron content of each genotype was measured in triplicate using inductively coupled plasma mass spectrometry (ICP-MS).

### Microscopy and Staining with DAPI, Perls/DAB and Toluidine Blue

Thin sections were first stained with DAPI in order to visualize nuclei (Roschzttardtz et al., 2013), the same section was stained by Perls/DAB, as described above. Then were stained with toluidine blue. Each slide was incubated in a 0.5% (w/v) solution of toluidine blue for 2 min and rinsed with distilled water. The samples were observed and photographed with a Nikon Eclipse 80i microscope.

## Results

### Iron Concentration and Distribution in Dry Seeds of Close Related Species to *Arabidopsis thaliana*

**FIGURE 1:**
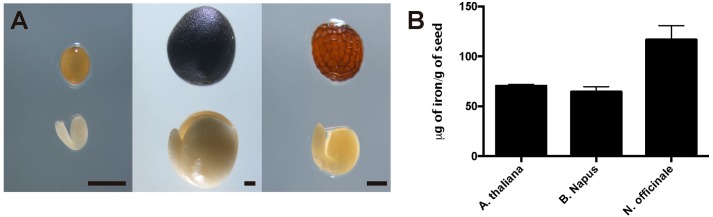
Iron concentration in Brassicaceae seeds. **(A)** Differences in size and form of Brassicales mature seeds and embryos used in this study. The left panel shows *Arabidopsis thaliana*, the middle panel shows *Brassica napus* and the right panel shows *Nasturtium officinale*. The bar scale represents 500 μm. **(B)** Iron content in mature seeds determined by ICP-MS. Values are the mean of three biological replicates (±SD).

To evaluate iron concentration and distribution in dry seeds from closely related Arabidopsis species, first we determine iron content in dry seeds from *Arabidopsis thaliana, Brassica napus* and *Nasturtium officinale* (**Figure 1A**). Dry seeds were isolated, and a microwave-assisted acid digestion was performed. **Figure 1B** show iron concentration of each species determined by inductively coupled plasma mass spectrometry (ICP-MS). Iron concentration in *Arabidopsis thaliana* and *Brassica napus* dry seeds (70.4 and 64.6 μg of iron/g of seeds, respectively) was lower compared to *Nasturtium officinale* (116.6 μg of iron/g of seeds), suggesting that there is no relation between iron concentration and seed size, as *Brassica napus* have the biggest seeds (**Figure 1A**) but also have a similar iron content as *Arabidopsis thaliana*.

**FIGURE 2:**
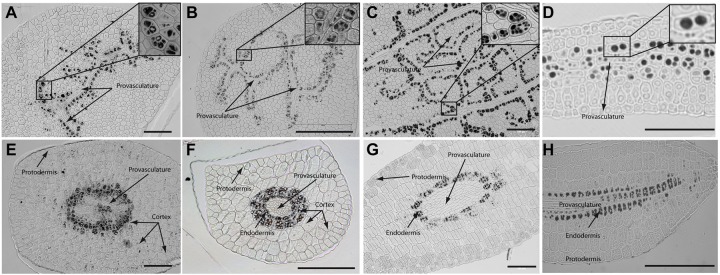
Iron distribution in *Brassica napus, Nasturtium officinale*, *Camelina sativa*, and *Arabidopsis thaliana* dry seed embryos. Embryos were dissected from dry seeds and thin sections were obtained and stained with Perls/DAB in order to reveal iron accumulation. *Brassica napus* sections are shown in **(A,E)**; *Nasturtium officinale* sections are shown in **(B,F)**; *Camelina sativa* sections are shown in **(C,G)** and *Arabidopsis thaliana* sections are shown in **(D,H)**. Cotyledons and hypocotyl are shown in **(A–D)** and **(E–H)** respectively. In panel A to D a zoom is shown in order to indicate iron accumulation into the vacuoles. Bars in panels correspond to 100 μm.

In order to determine where iron localizes in dry seed embryos from species closely related to *Arabidopsis thaliana*, we analyzed seven different plant species: *Brassica napus, Nasturtium officinale, Camelina sativa*, *Arabidopsis thaliana*, *Lepidium sativum, Brassica oleracea and Brassica oleracea* var. capitate (**Figure 2** and Supplementary Figure S2). Embryos from dry seeds were isolated and fixed. Thin sections of different embryo regions were analyzed, in particular, cotyledon and hypocotyl. **Figure 2** and Supplementary Figure S2 show Perls/DAB staining revealing iron distribution in embryos from these species. Cotyledons accumulate iron in vacuoles of cells surrounding provasculature (**Figures 2A–D** and Supplementary Figures S2A,B) as has been described before for *Arabidopsis thaliana* (Roschzttardtz et al., 2009). Provasculature complexity in cotyledon is clearly higher than Arabidopsis (Roschzttardtz et al., 2014, 2017). Iron accumulation was not observed in other cell types like protodermis and cortex cells in dry seed embryos (**Figures 2A–D** and Supplementary Figures S2A,B).

Interestingly, transversal sections of hypocotyl show differences in the number of cell layer where iron accumulates compared to Arabidopsis hypocotyl, where only one cell layer accumulates iron (Roschzttardtz et al., 2009). We found that at least two-cell layers accumulate iron in the hypocotyl of *Brassica napus, Nasturtium officinale*, *Camelina sativa, Lepidium sativum* and *Brassica oleracea* dry seed embryos (**Figures 2E–G** and Supplementary Figure S2C,D, respectively). Despite the differences in the number of cell layers where iron accumulates in dry seeds of *Brassica napus, Nasturtium officinale*, *Camelina sativa, Lepidium sativum* and *Brassica oleracea* compared to *Arabidopsis thaliana*, at subcellular level iron is stored in the vacuoles in cells surrounding provasculature (**Figures 2A–D** and Supplementary Figures S2A,B).

Analyses of embryonic root tips indicate that iron accumulates in different cells identified as endodermis-cortex cells (Supplementary Figure S3).

Supplementary Figure S1 shows the phylogenetic tree of the 5 species of Brassicaceae family used in this study. Interestingly, iron localization in vacuoles is a conserved trait in the analyzed plant species. As indicated above, differences were only observed in the number of cell layers that accumulate iron in the hypocotyls.

### Iron Distribution during *Brassica napus* Seed Development

As we showed, iron accumulates in vacuoles in embryos of seven species of Brassicaceae (**Figure 2** and Supplementary Figure S2). We used *Brassica napus* plants as model to study where iron localizes during seed development. *B. napus* produces bigger seeds compared with Arabidopsis plants allowing to study with more details different seed regions during its development (**Figure 1A**). In a phylogenetic point of view, *B. napus* is the more distant specie to *A. thaliana* used in this study. The fact that iron localizes in vacuoles of mature *B. napus* seeds is a strong indication that it is a conserved trait. Using *A. thaliana* as plant model it has been described that iron accumulates in embryo during seed maturation stages (Roschzttardtz et al., 2009), for this reason we analyzed three different stages of seed maturation, torpedo, bent cotyledon and mature stage before seed desiccation. In order to describe seed structures where iron accumulates before to be loaded into the embryo, we used in our analysis whole seeds including seed coat, endosperm and embryo. Whole seeds of *B. napus* containing embryos in different maturation stages as indicated above, were fixed and embedded in Technovit resin according to Section “Material and Methods.”

**FIGURE 3:**
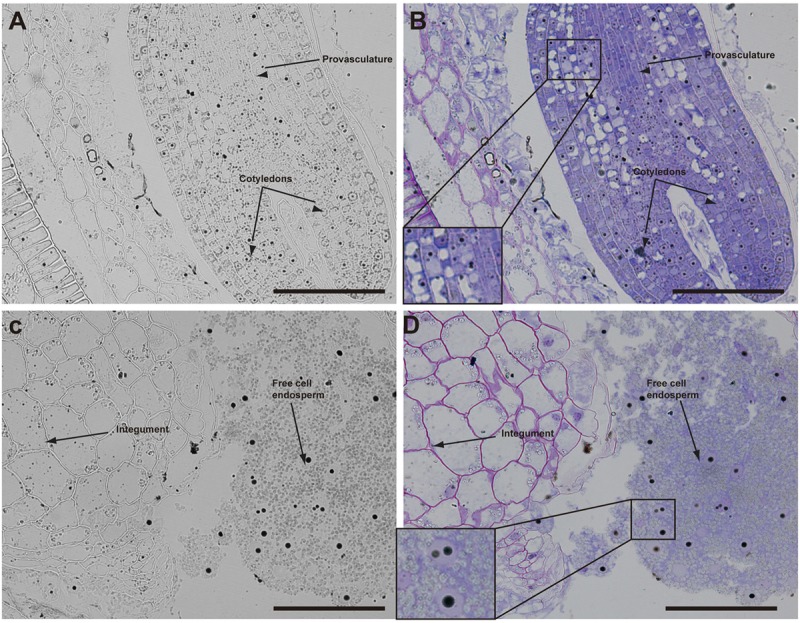
Iron distribution in *Brassica napus* seeds in torpedo embryo stage. *Brassica napus* seeds at torpedo stage dissected from siliques were embedded in Technovit resin and sectioned (3 μm) and then stained with Perls/DAB **(A–D)** and then with Toluidine blue **(B,D)**. In **(B,D)** a zoom is shown in order to indicate iron accumulation into the nuclei of the cells **(B)** and free cell endosperm **(D)**. The scale bar represents 100 μm.

The analysis of seeds with torpedo embryo stages revealed that iron localizes mainly in nuclei of integument cells, free cell endosperm and in different cell layers in the embryo (**Figure 3**). At this embryo stage, Perls/DAB and Toluidine blue staining show that in protodermal, cortex, endodermis and provasculature cells iron is localized in nuclei (**Figures 3A,B**). As vacuoles look empty after both staining, our results suggest that vacuoles of embryo cells are not an important compartment for iron storage at this developmental stage. Interestingly, strong iron staining was observed in nuclei (one to three iron pools were observed) of the free cell endosperm surrounding the embryo (**Figures 3C,D**).

**FIGURE 4:**
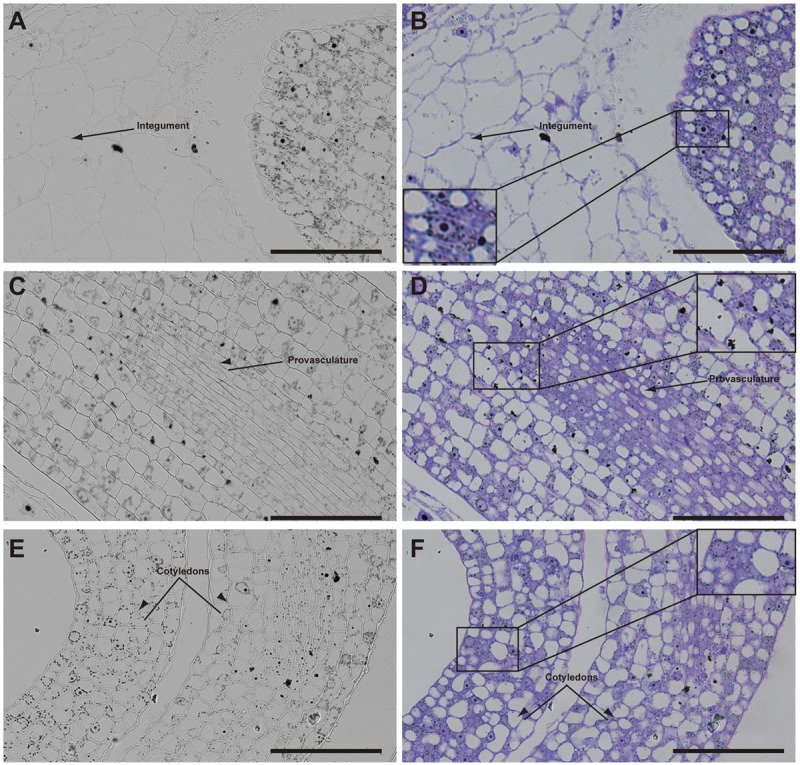
Iron distribution in *Brassica napus* seeds in bent cotyledon embryo stage. *Brassica napus* seeds at the bent cotyledon stage dissected from siliques were embedded in Technovit resin and sectioned (3 μm) and stained with Perls/DAB **(A–F)** and then with Toluidine blue **(D–F)**. In **(D–F)** a zoom is shown in order to indicate iron accumulation into and around the nuclei **(B)**, cellular structures surrounding the nuclei **(B,F)** and iron accumulation inside vacuoles **(D)**. The scale bar represents 100 μm.

Later, in seeds containing bent cotyledon embryo stage, integuments no longer contain iron inside the nuclei (**Figures 4A,B**). In the embryo, the number of cells containing iron in nuclei decreases. In embryonic root tips and cotyledons, iron is localized in structures surrounding the nuclei (**Figures 4A,B,E,F**), while vacuoles are starting to load iron in the hypocotyl endodermis cell layer (**Figures 4C,D**).

**FIGURE 5:**
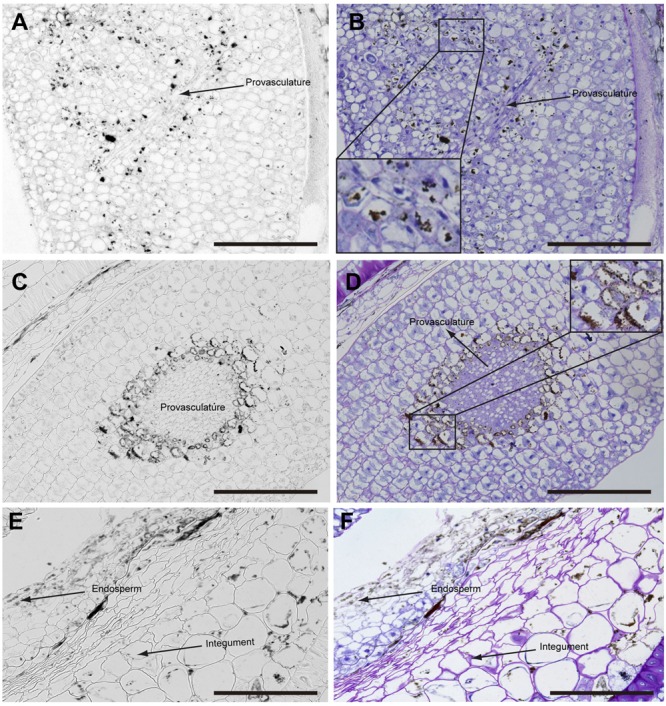
Iron distribution in *Brassica napus* seeds in mature embryo stage before seed desiccation. *Brassica napus* seeds at the mature embryo before seed desiccation stage dissected from siliques were embedded in Technovit resin and sectioned (3 μm) and stained with Perls/DAB **(A–F)** and then with Toluidine blue **(B,D,F)**. Cotyledons, hypocotyl and endosperm-seed coat are shown in **(A–F)**, respectively. In **(B,D)** a zoom are shown in order to indicate iron accumulation in the vacuoles. The scale bar represents 100 μm.

In mature seeds before desiccation, iron does not localize in nuclei (**Figure 5**) and vacuoles are the principal compartments for iron storage (**Figures 5B,D**). At this late stage of seed development, cells adjacent to provasculature load iron and protodermis and cortex cells have not detectable iron as the previous seed developmental stages (**Figure 5**).

In order to determine whether iron is accumulated in nuclei before torpedo stages, we use *N. officinale* developing embryos. Our results indicate that in the heart stage of embryo development iron is not detectable by Perls/DAB staining in embryo cells. This result strongly supports the conclusion that iron localizes in nuclei of embryo cells in the torpedo stage (Supplementary Figure S5). Similar stages of seed maturation were analyzed using *N. officinale*. No differences in iron distribution were observed compared to *B. napus* (Supplementary Figure S4).

### Iron Distribution at Subcellular Level during Seed Maturation

**FIGURE 6:**
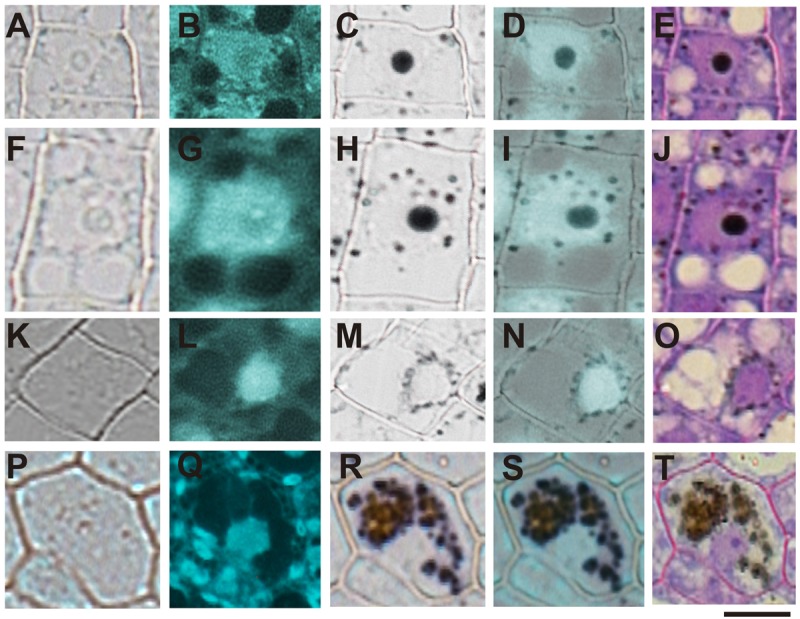
Iron transit across different subcellular compartments during seed development. Iron accumulates in nuclei of different cell type in early maturation stages of seed development **(A–E)**. In bent cotyledon stage, iron is detected in nuclei and cytoplasmic structures **(F–J)**, later is not longer detected in nuclei but accumulates only in cytoplasmic structures **(K–O)**, and finally in mature stages vacuoles of cells surrounding provasculature start to accumulate iron **(P–T)**. DAPI staining was used in order to visualize nuclei **(B,G,L,Q)**. Perls/DAB staining was used to reveal iron pools inside the cells **(C,H,M,R)**. Merge between DAPI and Perls/DAB staining are shown in **(D,I,N,S)** and Toluidine blue staining was used to reveal different cell structures **(E,J,O,T)**. **(A,F,K,P)** show controls cells without staining. The subsequent staining was performed over the same sections. The scale bar represents 20 μm.

In order to confirm the subcellular compartments accumulating iron during development we performed consecutively staining with DAPI, Perls/DAB and toluidine blue on the same sections of different developmental stages of *B. napus* embryos. Iron accumulates in nuclei of different cell types in early maturation stages of seed development (**Figures 6A–E**). In bent cotyledon stage, iron is not longer detected in nuclei and accumulates in cytoplasmic structures (**Figures 6K–O**). Finally in mature stages, vacuoles of cells surrounding provasculature start to accumulate iron (**Figures 6P–T**). Details of unique cells are shown in **Figure 6**, indicating unambiguously were iron localizes in different cases, nuclei, cytoplasmic structures or vacuoles.

## Discussion

During seed development embryo accumulates nutrients that will be used in the transition of heterotrophy to autotrophy metabolism during germination and post germination stage of development. In the case of iron, the principal transporter expressed in root involved in iron acquisition in Arabidopsis *IRT1* is expressed 3–4 days after germination (Lanquar et al., 2010). Before roots are able to acquire iron from the soil, iron that was stored during seed development is remobilized from vacuoles of endodermis and bundle sheath cells (Roschzttardtz et al., 2009; Lanquar et al., 2010; Mary et al., 2015). When iron is not correctly localized as in *vit1* mutants or not remobilized as in *nramp3 nramp4* double mutant, germinated seeds have difficulties to growth in iron deficiency conditions (Kim et al., 2006; Lanquar et al., 2010; Mary et al., 2015). This indicates that iron storage and localization in embryos is pivotal for seed physiology (Kim et al., 2006).

In this article, different stages of seed maturation were analyzed by a histochemical approach (Perls/DAB staining) in order to reveal iron localization. Perls/DAB method is specific and very sensitive for iron detection and can detect both Fe^2+^ and Fe^3+^ forms (Roschzttardtz et al., 2009). Other methods more quantitative have been used to reveal iron localization in seeds, indicating that an Arabidopsis embryo contains a fraction of the total iron of a seed (Schnell Ramos et al., 2013). Despite of not being a quantitative method, Perls/DAB staining allows to obtain information at subcellular level of any plant tissues and stages of plant development (Roschzttardtz et al., 2013).

**FIGURE 7:**
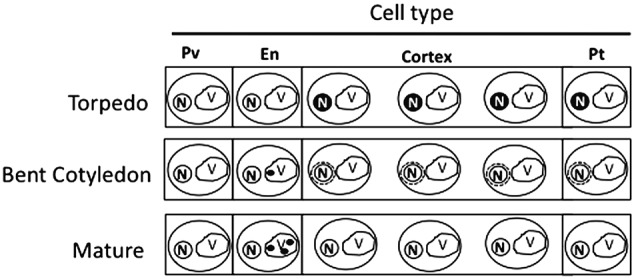
Model of iron distribution during seed development. At the torpedo stage iron accumulates mainly in nuclei of different cell types. In bent cotyledon stage, iron is not longer detected in nuclei and accumulates in unknown cytoplasmic structures (possibly endoplasmic reticulum or mitochondria), later in mature stages vacuoles of cells surrounding provasculature start to accumulate iron. Pv, provasculature; En, endodermis; Pt, protodermis. N, nuclei; V, vacuole.

Our analysis showed that iron was not localized in an unique subcellular compartment during seed development. In early maturation stages (embryo in torpedo stage) iron was localized principally in nuclei of integument, endosperm and embryo cells. This result is very surprising, indicating that vacuoles are not the only relevant subcellular compartment for iron storage during seed development (**Figures 3, 4**). Interestingly, Perls/DAB did not detect iron in embryo at heart stage, suggesting strongly that iron is accumulated in nuclei not before torpedo stages (Supplementary Figure S5). Later iron seems to be gradually concentrated in structures surrounding nuclei (possibly mitochondria or endoplasmic reticulum) before to be loaded into the vacuoles of endodermis cells (**Figure 5**). The micronutrient manganese has been detected in endoplasmic reticulum during seed development, but more studies may be performed in order to identify the nature of the structures where iron is detected before to be mobilized to the vacuoles (Otegui et al., 2002). A model of iron dynamic during embryo loading is shown in **Figure 7**. The presence of iron in nuclei of pea embryos was previously reported in Roschzttardtz et al. (2011), however, in this present study we shown that iron localizes in nuclei only during torpedo stages in Brassicaceae seeds. These results open interesting questions about the role of nuclei at this embryo stage of development. Our results suggest that nuclei may be a reservoir of iron during seed development or that iron may have unknown functions in nuclei of embryos in torpedo stage. Very recently, a specific role of iron in promoting meristematic cell division in adventitious root formation has been proposed (Hilo et al., 2017), however, the molecular role of iron in nuclei remains unknown.

Regarding iron distribution, we found that species closely related to *Arabidopsis thaliana* have some differences in dry seed embryos. For instance, iron is accumulated into the vacuoles of two-cell layer in mature embryos of *Lepidium sativum* showing a clear difference with Arabidopsis mature embryos, where only one cell layer accumulates iron (**Figure 2**; Roschzttardtz et al., 2009). A study reporting iron localization in dry seeds of 13 genoypes of three Phaseolus species has shown that large concentrations of iron are accumulated in the cytoplasm of cells surrounding the provascular bundles (Cvitanich et al., 2010). In the case of monocots, the highest iron concentration occurs in scutellum and outer regions of the embryo while a very low Fe is detected in the outermost layers of the endosperm and the single-layered aleurone that surrounds the endosperm (Johnson et al., 2011). This suggests that different mechanisms of iron acquisition may play important roles in different seed species. In our group, we are interested in elucidating the molecular mechanisms that may explain these differences.

## Author Contributions

MI, SG-G, NN, and HR performed experiments and designed the research. MP was involved in the phylogenetic tree analysis. All authors participated in manuscript preparation.

## Conflict of Interest Statement

The authors declare that the research was conducted in the absence of any commercial or financial relationships that could be construed as a potential conflict of interest.
