# Supplementary figures and images for: Dynamic Subcellular Localization of Iron during Embryo Development in Brassicaceae Seeds
Source: Front Plant Sci. 2017 Dec 22;8:2186. doi: 10.3389/fpls.2017.02186 (PMC5744184; doi:10.3389/fpls.2017.02186)

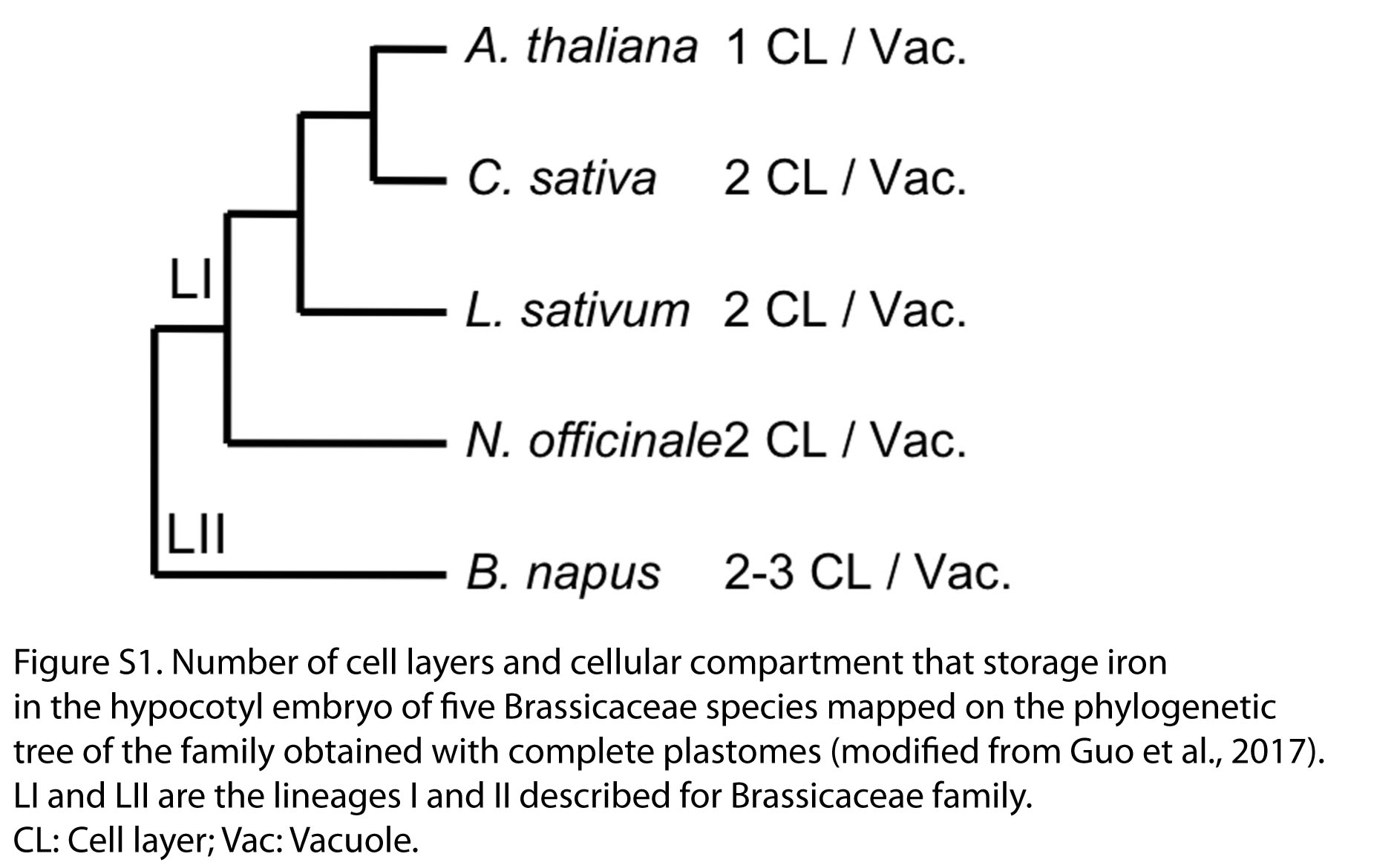

Supplement: Supplementary file 1 [file Image_1.jpg]

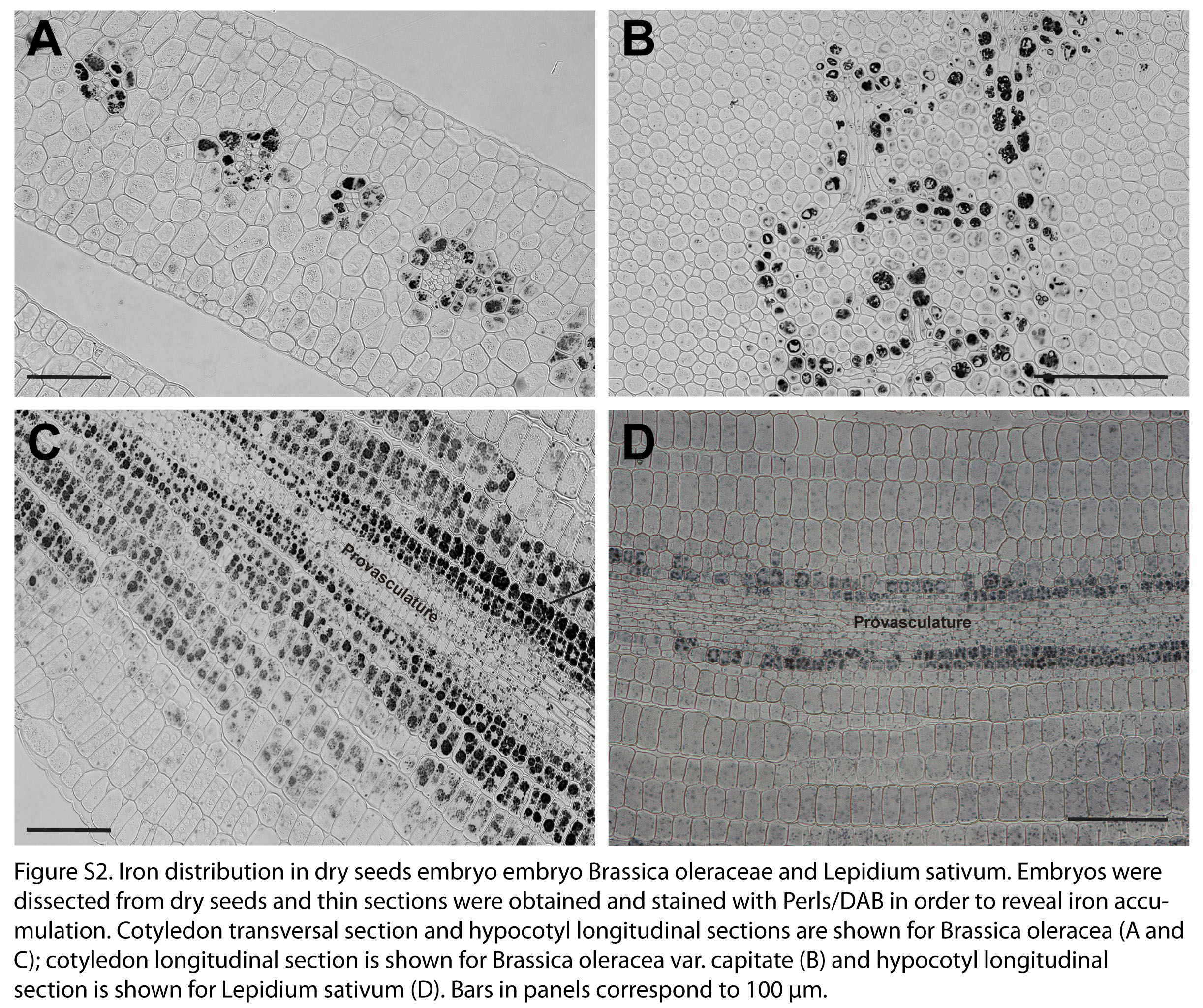

Supplement: Supplementary file 2 [file Image_2.jpg]

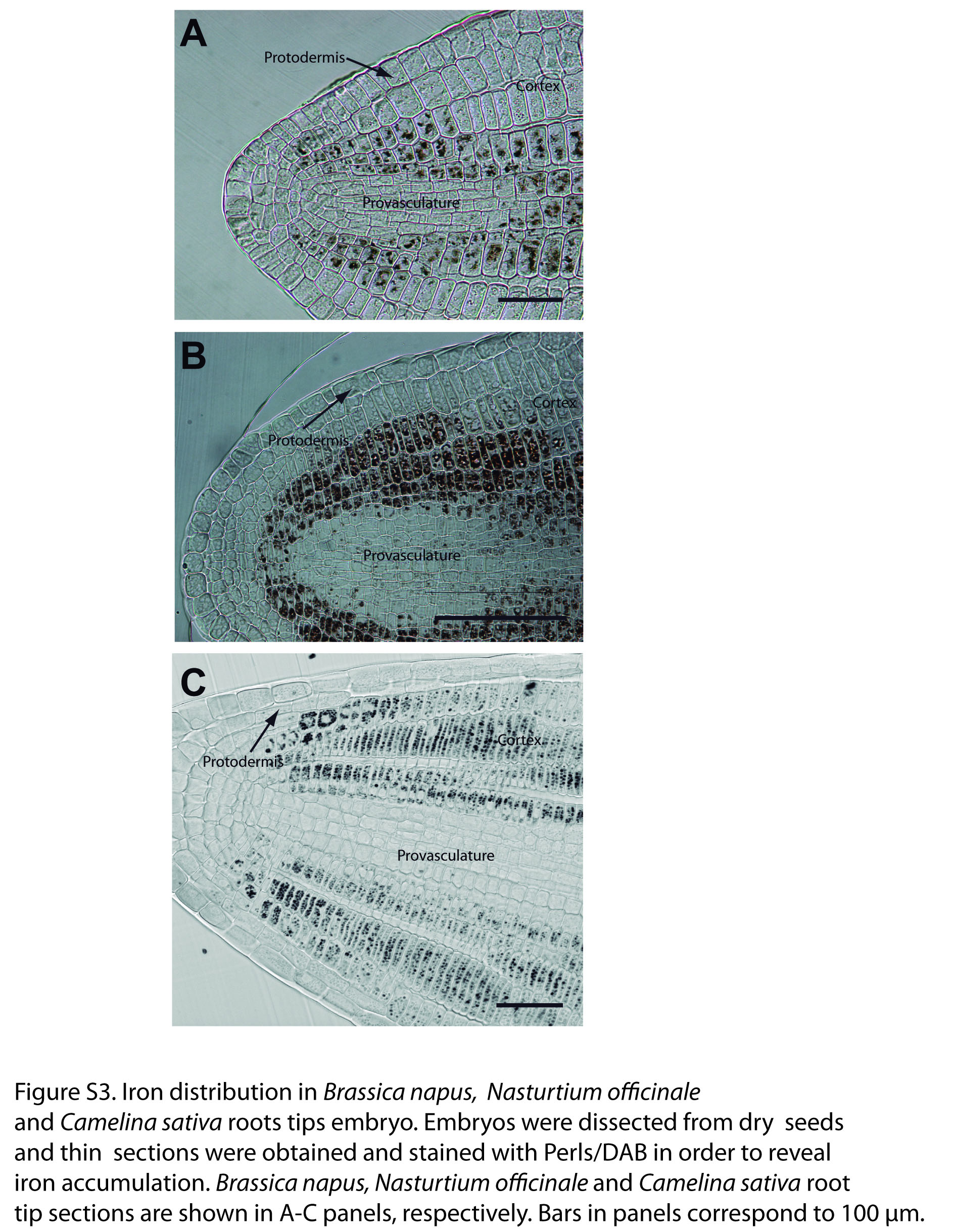

Supplement: Supplementary file 3 [file Image_3.jpg]

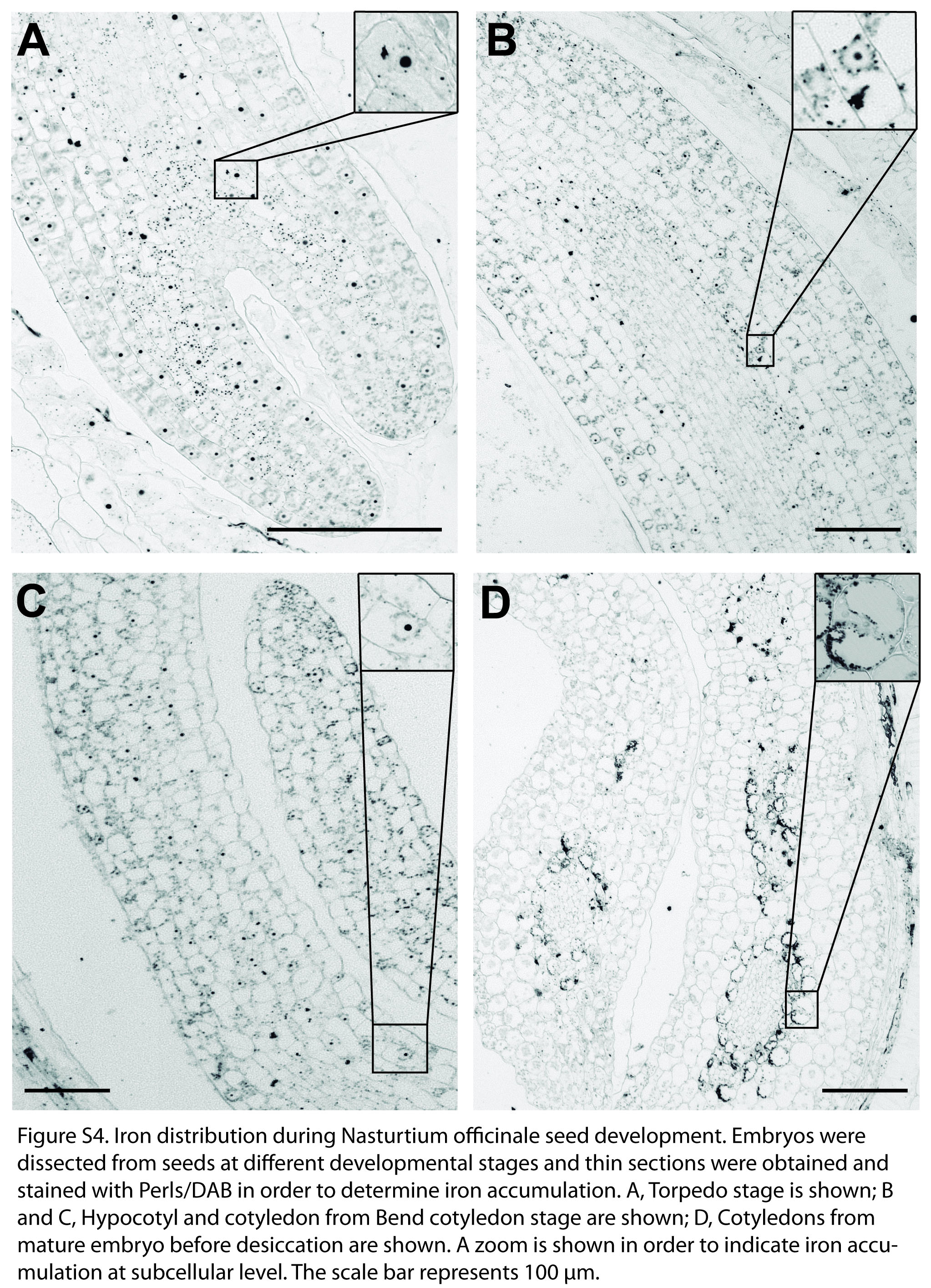

Supplement: Supplementary file 4 [file Image_4.jpg]

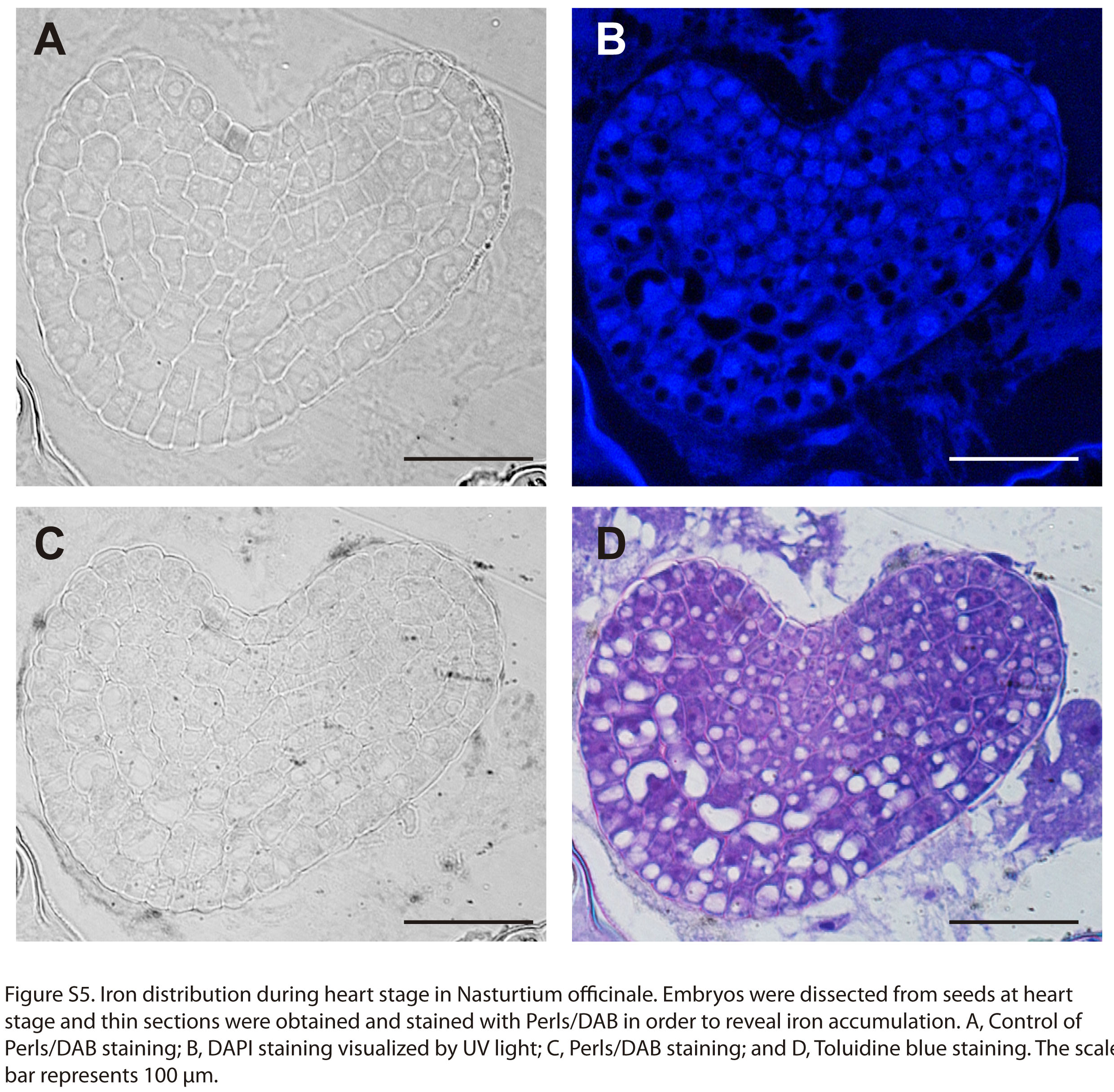

Supplement: Supplementary file 5 [file Image_5.jpg]
